# The E3-Ubiquitin Ligase TRIM50 Interacts with HDAC6 and p62, and Promotes the Sequestration and Clearance of Ubiquitinated Proteins into the Aggresome

**DOI:** 10.1371/journal.pone.0040440

**Published:** 2012-07-09

**Authors:** Carmela Fusco, Lucia Micale, Mikhail Egorov, Maria Monti, Ester Valentina D’Addetta, Bartolomeo Augello, Flora Cozzolino, Alessia Calcagnì, Andrea Fontana, Roman S. Polishchuk, Gerard Didelot, Alexandre Reymond, Piero Pucci, Giuseppe Merla

**Affiliations:** 1 Medical Genetics Unit, IRCCS Casa Sollievo Della Sofferenza Hospital, San Giovanni Rotondo, Italy; 2 Telethon Institute of Genetics and Medicine, Naples, Italy; 3 CEINGE Advanced Biotechnology and Department of Organic Chemistry and Biochemistry, Federico II University, Napoli, Italy; 4 Unit of biostatistics, IRCCS Casa Sollievo Della Sofferenza Hospital, San Giovanni Rotondo, Italy; 5 Center for Integrative Genomics, University of Lausanne, Lausanne, Switzerland; Boston University Medical School, United States of America

## Abstract

In this study we report that, in response to proteasome inhibition, the E3-Ubiquitin ligase TRIM50 localizes to and promotes the recruitment and aggregation of polyubiquitinated proteins to the aggresome. Using *Hdac6*-deficient mouse embryo fibroblasts (MEF) we show that this localization is mediated by the histone deacetylase 6, HDAC6. Whereas *Trim50*-deficient MEFs allow pinpointing that the TRIM50 ubiquitin-ligase regulates the clearance of polyubiquitinated proteins localized to the aggresome. Finally we demonstrate that TRIM50 colocalizes, interacts with and increases the level of p62, a multifunctional adaptor protein implicated in various cellular processes including the autophagy clearance of polyubiquitinated protein aggregates. We speculate that when the proteasome activity is impaired, TRIM50 fails to drive its substrates to the proteasome-mediated degradation, and promotes their storage in the aggresome for successive clearance.

## Introduction

The ubiquitin proteasome system (UPS) is a highly conserved pathway that removes non-functional, damaged, and/or misfolded proteins from the cell. However, when the capacity of the proteasome is impaired, misfolded proteins cannot be properly cleared and they accumulate into the aggresome [1], [2], an inclusion body localized in the proximity of the microtubule-organizing centre (MTOC) [3], [4]. Microtubule-associated histone deacetylase 6 (HDAC6) mediates this process [5]. Through its ubiquitin-binding BUZ finger domain, HDAC6 binds to and facilitates the transport of polyubiquitinated misfolded proteins along microtubules to aggresome [2]. Aggresome clearance is mediated by ubiquitin-binding proteins like p62/SQSTM1 and NBR1 [6]. These adaptor proteins through their ubiquitin-binding domain (UBA) decide the fate of protein degradation either through UPS or autophagy-lysosome pathway [6], [7], [8]. Ubiquitin ligases are terminal enzyme in the process of ubiquitination, which provides specificity to the pathway by recognizing the substrates. Experimental evidences suggest that E3-Ubiquitin ligases play an important role also in the execution of autophagy [9], [10]. Therefore searching for new E3-Ubiquitin ligases involved in such processes are of interest.

TRIM proteins are RING E3-Ubiquitin ligases defined by the presence of a tripartite motif consisting of a RING, one or two B-Box, and a Coiled-Coil domain involved in a variety of cellular processes, including regulation of cell cycle progression, differentiation, development, oncogenesis, and apoptosis [11], [12], [13]. *TRIM50* is one of 28 hemizygous genes mapping to the region rearranged in Williams Beuren syndrome (WBS) [14], [15], a genomic disorder characterized by mental retardation and multiple dysmorphic and metabolic features [16]. TRIM50 encodes an E3-Ubiquitin ligase that self-associates to form cytoplasmic bodies in the cell, like other TRIM proteins [13], [15]. The nature and role of these bodies as well as the cellular function of TRIM50 is just beginning to emerge [15]. Here we report that TRIM50 cytoplasmic bodies are aggresome precursors. We show that during proteasome impairment TRIM50 promotes the recruitment and aggregation of polyubiquitinated proteins to the aggresome, and participates to aggresome clearance. In addition we identified two novel TRIM50 protein interactors, HDAC6 and p62, and show that TRIM50 determines the accumulation of both p62 and HDCA6 into an insoluble protein aggregate fraction.

**Figure 1:**
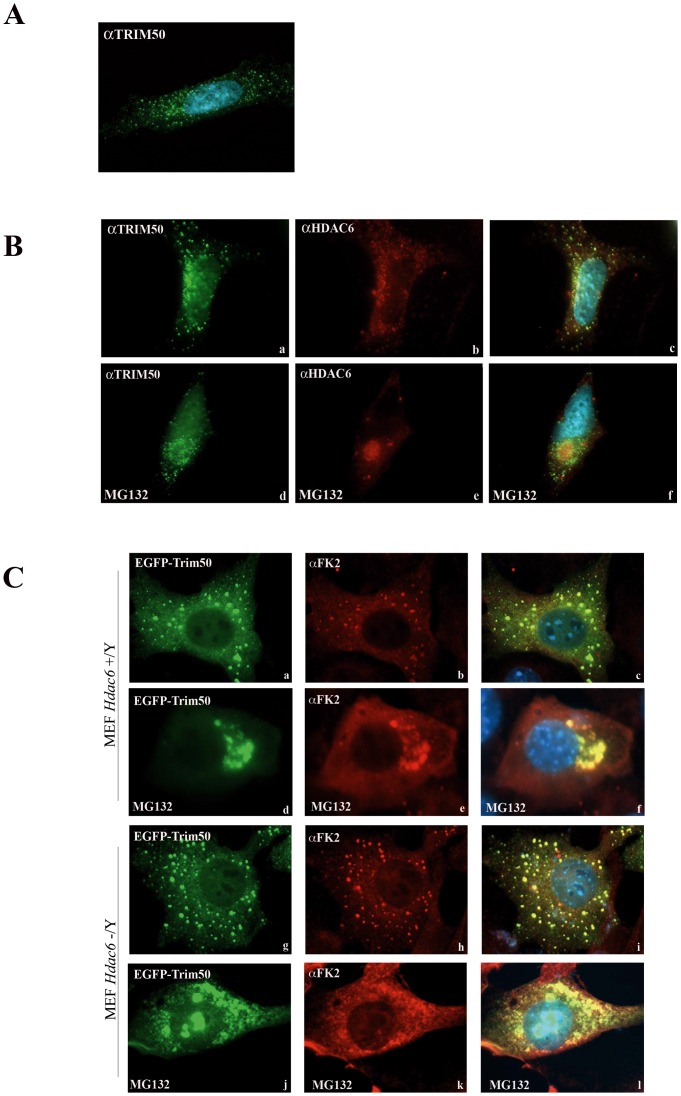
TRIM50 bodies colocalize into aggresome by a Hdac6-dependent way. (A) SH-SY5Y cells were stained with anti-TRIM50 antibody. (B) SH-SY5Y cells were stained with anti-TRIM50 and anti-HDAC6 antibodies. The cells were treated with 25 µM MG132 for 6 h (d,e,f). (C) Hdac6 wild type and Hdac6 deficient mouse fibroblasts were transfected with EGFP-Trim50 followed by treatment with 25 µM MG132 for 6 h where indicated, and immunostained with FK2 antibody.

## Results

### TRIM50 Localizes to Cytoplasmic Bodies

We previously reported that ectopically expressed TRIM50 localizes mainly into discrete cytoplasmic punctuate structures heterogeneous in size and shape, with the intact central region of the protein (B-Box and Coiled-Coil domains) indispensable for the proper localization [15]. To rule out that the observed pattern was due to *TRIM50* overexpression, we showed that also the endogenous TRIM50 localizes in diffuse cytoplasmic round bodies in human neuroblastoma-derived SH-SY5Y cell lines (Figure 1A). Ectopically expressed TRIM50 cytoplasmic bodies did not associate with known cellular compartments and markers including trans- and cis-Golgi, endosomes, caveolae, vesicles, lysosomes, cytosckeletal structures, peroxisomes, stress granules, and P-bodies (Figure S1).

**Figure 2:**
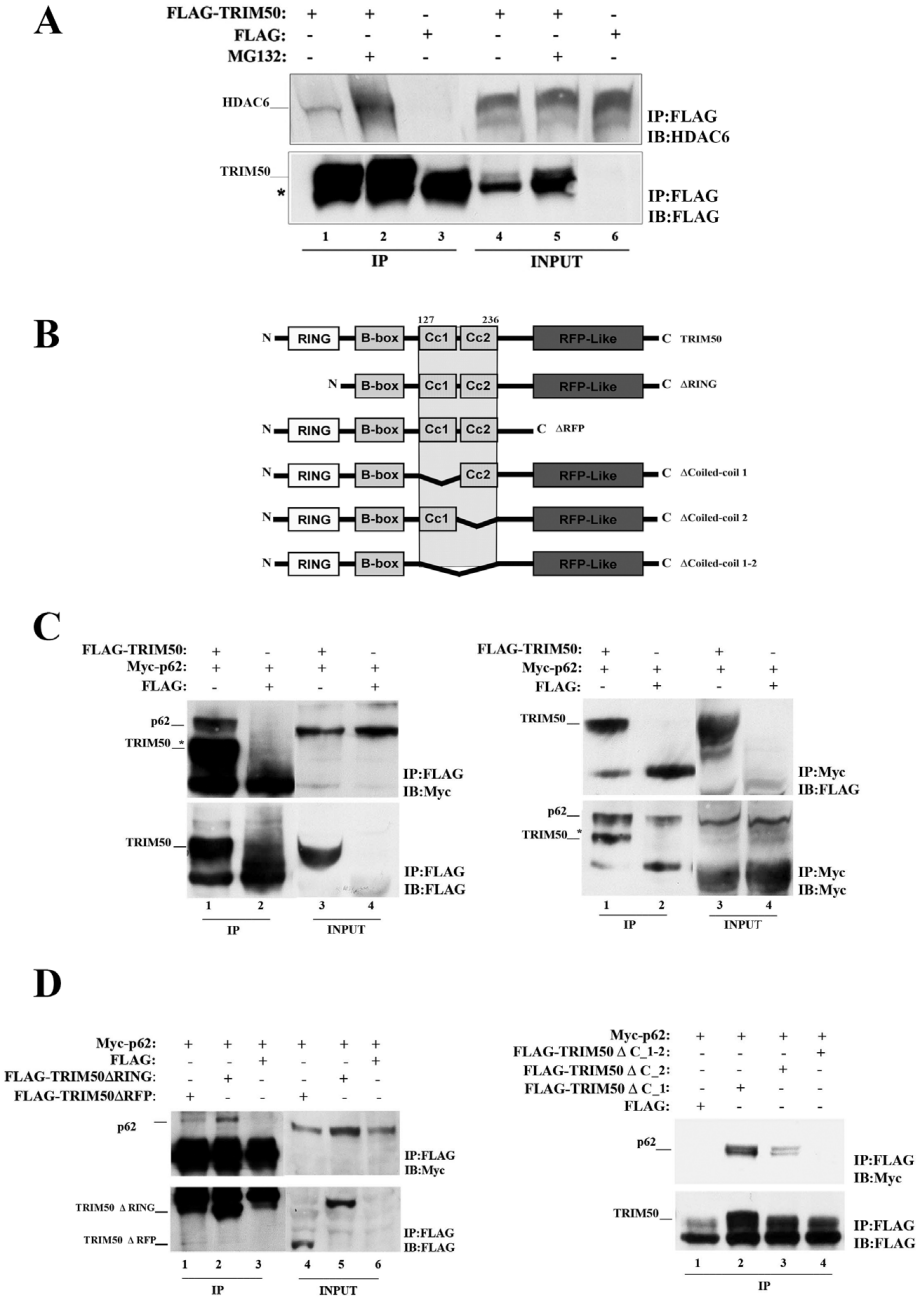
TRIM50 interacts with HDAC6 and p62. (A) The interaction between TRIM50 and HDAC6 was assayed in HEK293 FLAG-TRIM50#3 cell line, treated with MG132. The cell lysates were immunoprecipitated (IP) with anti-FLAG and Immunoblot (IB) with HDAC6 antibody. Asterisk indicate IgG aspecific band. (B) Schematic representation of TRIM50 deletion mutants used, with the minimal TRIM50 interaction region. (C) The interaction between TRIM50 and p62 was assayed in FLAG-TRIM50#3 transfected with Myc-p62. The cell lysates were immunoprecipitated with anti-FLAG (left) and with anti-Myc (right) antibodies, respectively. Immunoblot were done as indicated (asterisk indicates FLAG-TRIM50 coming from the first FLAG blotting). (D) Total lysates of HEK293 cells transfected both with TRIM50 deletion mutants and Myc-p62 were immunoprecipitated with anti-FLAG and immunoblotted with anti-Myc.

Using live microscopy, we showed that ectopically expressed TRIM50 cytoplasmic bodies are highly motile structures varying in size and shape that exhibit multidirectional short and fast jumping movements and able to assemble larger cytoplasmic bodies from smaller particles (Figure S2A–B, and Movie S1). To gain insight into the dynamics of TRIM50 bodies, we performed Fluorescence Recovery After Photobleaching (FRAP) [17]. Our analysis revealed that the fluorescence of a photobleached cytoplasmic body significantly recovers within the 2 min of the time-period experiment (Fig. S2C, and Movie S2). The FRAP data confirm that TRIM50 cytoplasmic bodies are dynamic, rapidly exchanging between different cytoplasmic regions, and promptly turned over.

**Figure 3:**
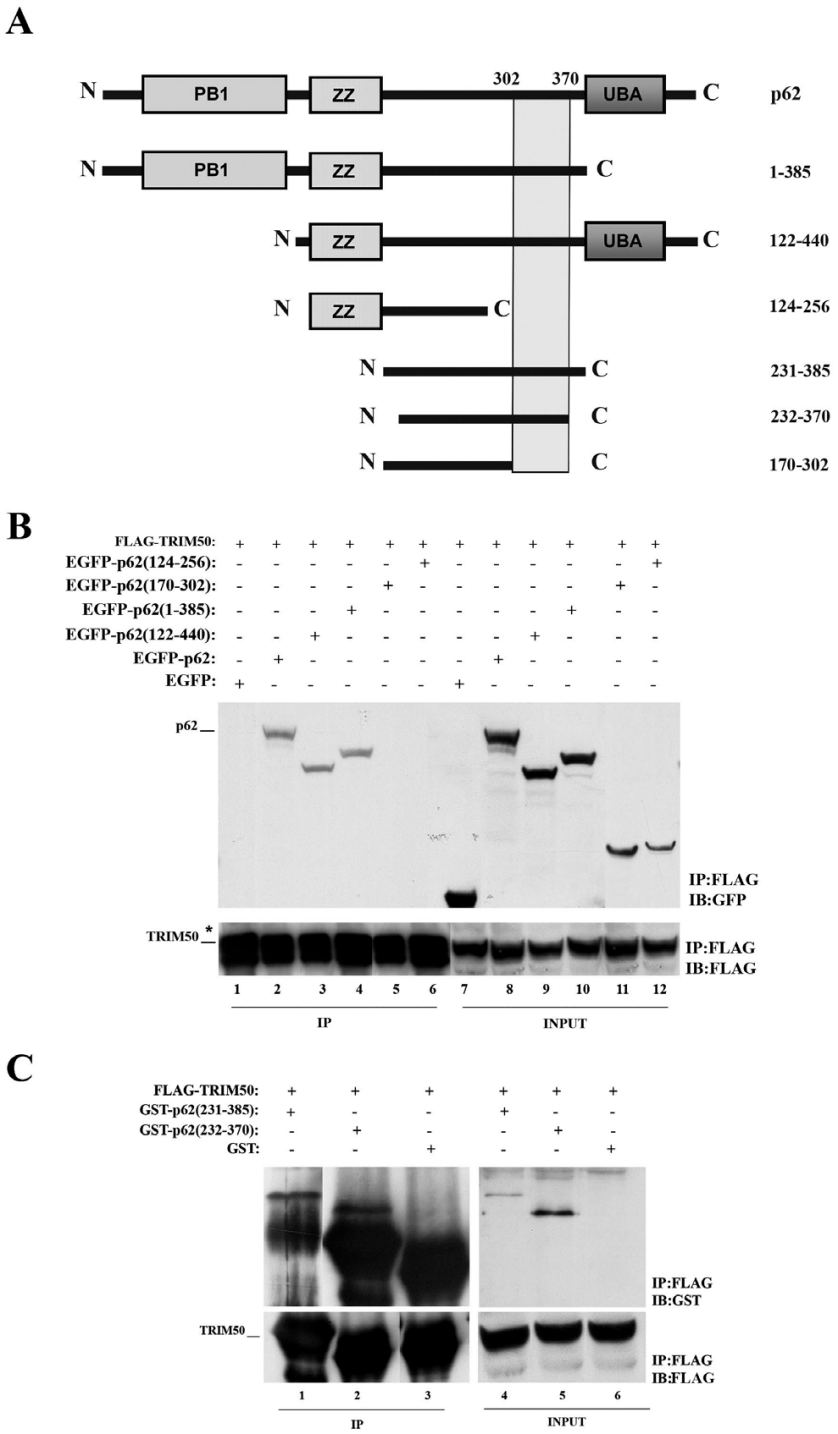
p62 deletion mutants interacting with TRIM50. (A) Schematic representation of p62 deletion mutants with the minimal region of interaction with TRIM50. Lysates from FLAG-TRIM50#3 expressing a number of p62 deletion mutants were immunoprecipitated with anti-FLAG and immunoblotted with anti-GFP (B) and anti-GST (C).

Further characterization of TRIM50 bodies was achieved by Correlative Light-Electron Microscopy (CLEM). This analysis revealed that the fluorescent bodies corresponded to heterogeneous in morphology TRIM50-containing protein aggregates, confirming their tendency to self-associate into larger structures (Figure S2D).

### TRIM50 Associates with Aggresome

We asked whether TRIM50 bodies associate with aggresome. In SH-SY5Y cells, treated with the proteasome inhibitor MG132 and stained with FK2, which recognizes polyubiquitinated proteins, endogenous TRIM50 concentrated close to a perinuclear structure whose morphology and localization resemble that of aggresome (Figure S3A, d–f). To further investigate the possible link between TRIM50 and aggresome, we used HDAC6, an established aggresome marker [18]. We found that both endogenous and transfected TRIM50 partially located with HDAC6 under proteasome inhibition (Figure 1B, d–f; Figure S3B, d–f). This cellular localization does not depend on the E3-ligase activity of the RING domain of TRIM50 as a mutant lacking the RING domain retains the ability to localize to aggresome (Figure S4A).

**Figure 4:**
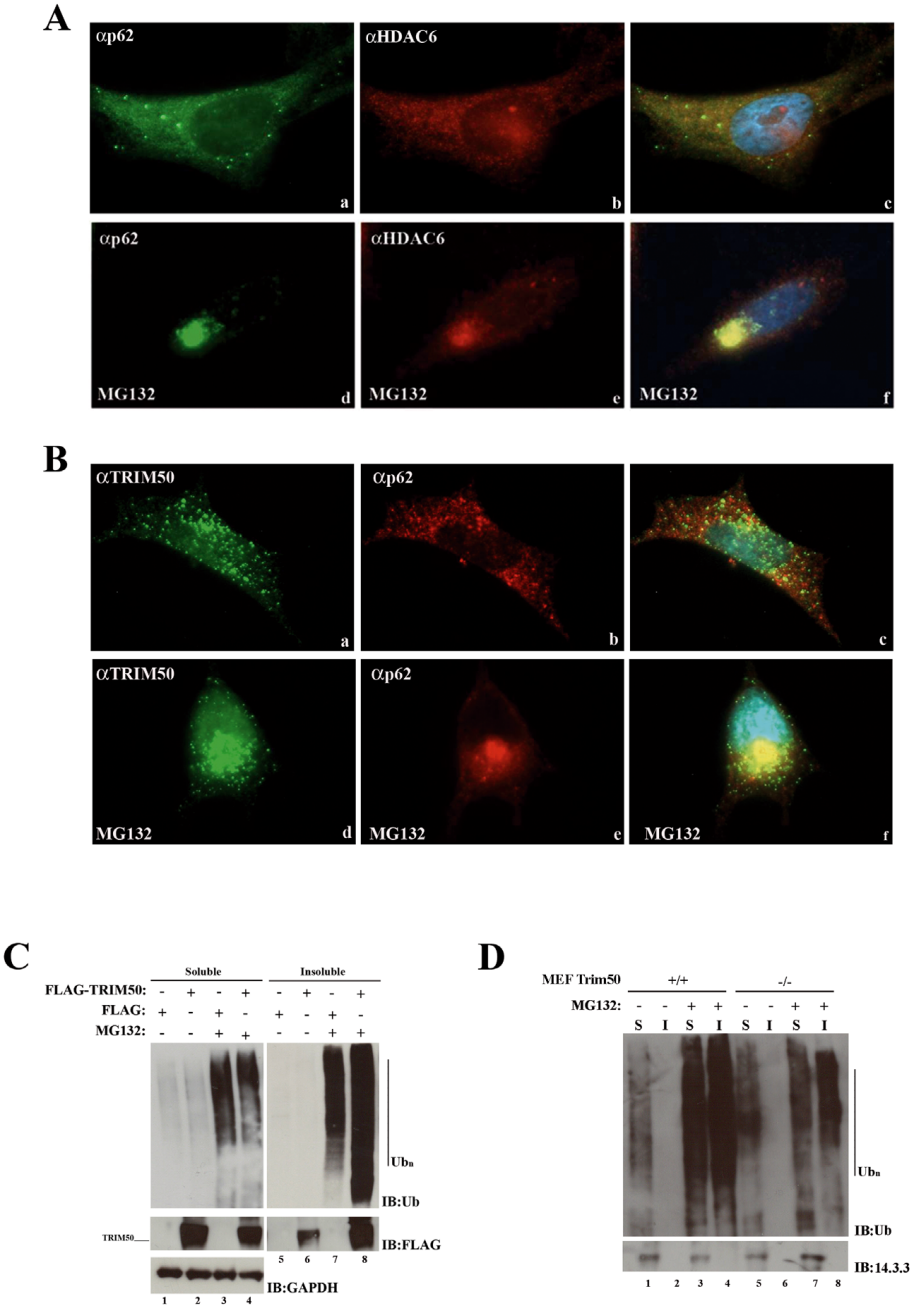
TRIM50 colocalizes with p62 into aggresome. (A) SH-SY5Y cells were stained with anti-HDAC6 and with anti-p62 antibodies. The cells were treated with 25 µM MG132 for 6 h (d,e,f). (B) SH-SY5Y cells were stained with anti-TRIM50 and anti-p62 antibodies. The cells were treated with 25 µM MG132 for 6 h (d,e,f). (C) Lysates from FLAG-TRIM50#3 and FLAG#3 cell lines, treated with vehicle (–) or MG132 (+) were separated in RIPA detergent-soluble (S) and detergent insoluble (I) fractions and immunoblotting with anti-FLAG and anti-ubiquitin antibodies. (D) Lysates from MEF *Trim50* cell lines with different genotype (+/+, −/−), treated with vehicle (–) or MG132 (+) were separated in RIPA detergent-soluble (S) and detergent insoluble (I) fractions, analyzed by Western Blot and immunoblotting with anti-ubiquitin antibody.

In accordance with the central role of retrograde microtubule-dependent transport in the formation of aggresome, nocodazole treatment of SH-SY5Y cells prevented the localization of TRIM50 to aggresome (data not shown). In agreement we demonstrated that TRIM50 interacts with Tubulin beta 2B class IIb (Tubb2b) (NM_178012.4), a microtubules component (Figure S3C). Together these findings suggest that TRIM50 bodies may represent aggresome precursors that in response to proteasome inhibition move towards aggresome by a microtubule dependent transport.

**Figure 5:**
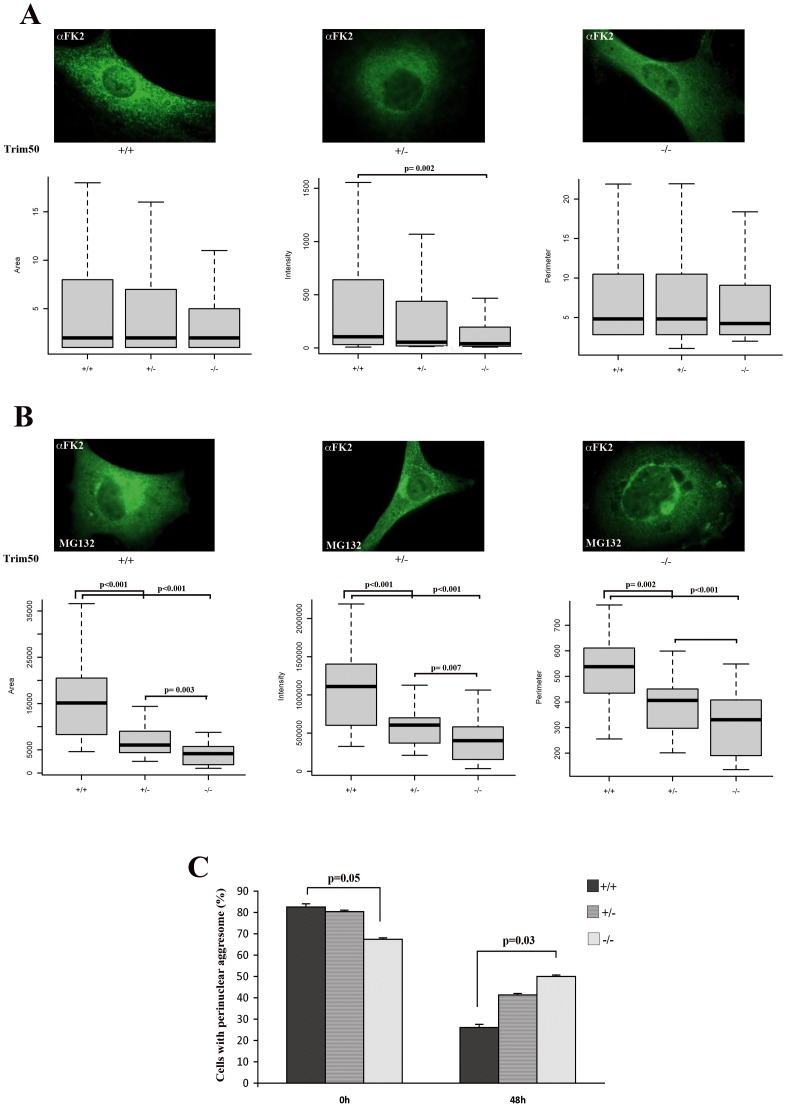
TRIM50 promotes the accumulation of polyubiquitinated proteins into the aggresome. (A) MEF *Trim50* cell lines were stained with anti-FK2 antibody. The area, perimeter and intensity of signal of three independent experiments of each genotype were estimated using imageJ program. (B) MEF *Trim50* cell lines were treated with 10 µM MG132 over night and stained with anti-FK2 antibody. The area, perimeter and intensity of signal of three independent experiments of each genotype were estimated using imageJ program. (C) MEF *Trim50* cell lines were treated with 10 µM MG132 over night, incubated o and 48 h with DMEM after MG132 wash out and stained with anti-FK2 antibody. The diagram shows the percentage of aggresome-positive cells.

To assess whether HDAC6 is required for the proper localization of TRIM50, we performed immunofluorescence assays in *Hdac6* deficient mouse fibroblasts [19]. We found that in *Hdac6* wild-type cells, Trim50 bodies localize within FK2-ubiquitin-containing aggresomes upon MG132 treatment (Figure 1C, d–f). Conversely, when MG132 was added to *Hdac6* knock out cells Trim50 bodies were unable to form whole aggresome, although they still continued to partially colocalize with ubiquitinated aggregates (Figure 1C, j–l). These results indicate that HDAC6 is required for the proper localization of TRIM50 bodies and of ubiquitinated proteins within the aggresome. We then investigated whether the observed TRIM50-HDAC6 colocalization results also in their physical interaction. As no anti-TRIM50 antibodies are actually effective for immunoprecipitation assays, we generated a HEK293 cell line that stably expresses a FLAG-tagged TRIM50 (hereafter referred to as FLAG-TRIM50#3). As shown in Figure 2A, TRIM50 interacts with endogenous HDAC6; an interaction that strengthens in response to MG132 treatment (compare lane 1 to lane 2 in Figure 2A).

**Figure 6:**
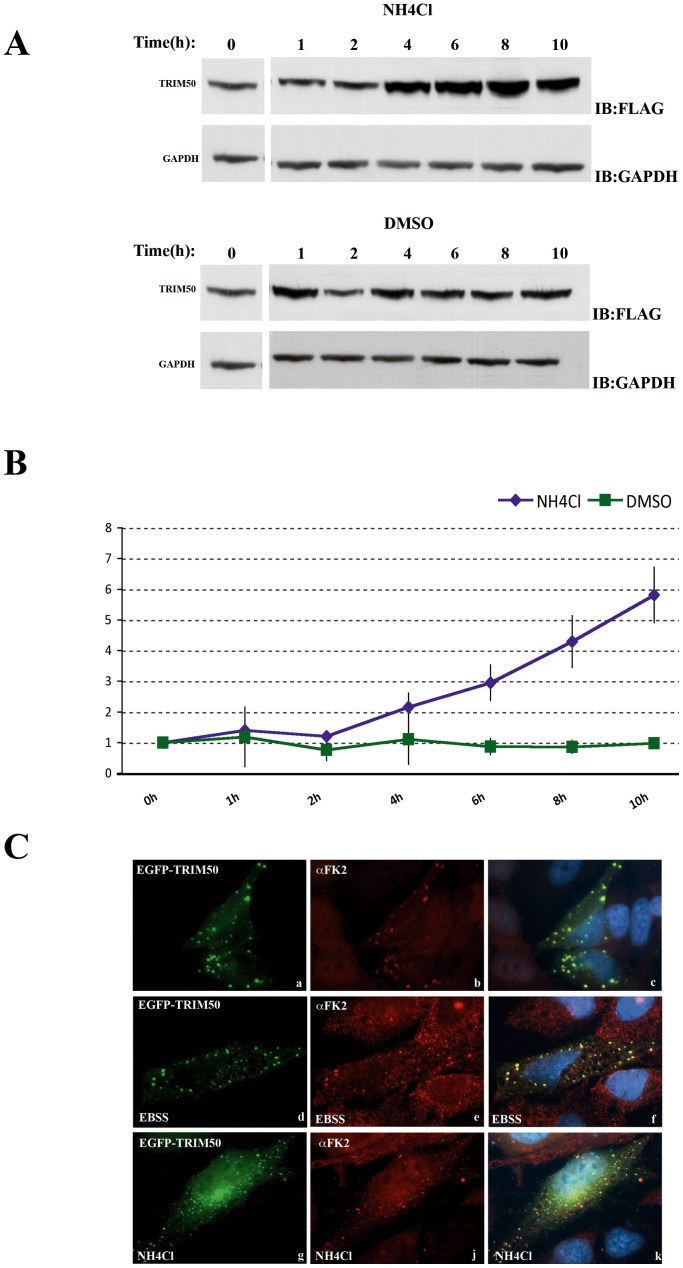
TRIM50-related aggresome and TRIM50 itself are partially degraded through the autophagy-lysosomal pathway. (A) FLAG-TRIM50#3 cells were treated with 20 mM NH_4_Cl (A) and DMSO for a period of 10 hours. Cells were lysed at the indicated time after the initiation of treatment and analysed by Western blotted using FLAG and GAPDH antibodies, respectively. The experiments were performed three times and typical results are shown. (B) Curves describing TRIM50 levels as a function of time, based on the results in panel A. The density of each band was determined by densitometer. The half-time of TRIM50 was determined by calculating the protein level at each time, normalized to the corresponding GAPDH level, to the initial amount of TRIM50 protein and compared with the control cells treated with DMSO. (C) HeLa cells overexpressing EGFP-TRIM50 were incubated in complete medium (a–c), EBSS (d–f), and DMEM supplemented with NH_4_Cl 20 mM for 2 h respectively and stained with an anti-FK2 antibody.

### TRIM50 Interacts with p62

Proteomics assays were designed to identify novel TRIM50 partners. TRIM50 complexes were isolated by immunoprecipitation of total protein lysate from FLAG-TRIM50#3 cells and individual protein components were solved and identified by nano LC-MS/MS. Among the putative TRIM50 interactors, we focused on p62 (also known as Sequestosome 1/p62), because of its involvement in the formation of protein aggregates [20], its role as shuttling factor for the delivery of polyubiquitinated substrates to the proteasome [21], and for emerging central importance at the intersection of proteasome and autophagy pathways [22]. First we assessed whether TRIM50 and p62 self associate. FLAG-TRIM50#3 cells was transfected with a plasmid that expresses Myc-tagged p62. Total cell lysates were then immunoprecipitated with an anti-FLAG and immunoblotted with an anti-Myc specific antibody. An anti-Myc reactive band was exclusively precipitated in the presence of FLAG-TRIM50 (Figure 2C, left side). Consistently, we detected FLAG-TRIM50 in protein lysates immunoprecipitated with an anti-Myc and immunoblotted with an anti-FLAG antibody (Figure 2C, right side), substantiating the interaction between TRIM50 and p62.

Next, to map the TRIM50 domain(s) involved in p62 interaction, we used a set of FLAG-TRIM50 mutants [15] (Figure 2B). Upon co-transfection with Myc-p62 and coimmunoprecipitation with an anti-FLAG, we found that both coiled coil domains of TRIM50 are required for the efficient binding of p62 (Figure 2D). Likewise, to define the p62 interaction region, we performed co-immunoprecipitation and GST pull down assays using p62 deletion mutants (Figure 3A). As reported in Figure 3B and 3C we narrowed the p62 interaction region between aminoacids 302–370, a region that includes the LC3 binding region [23].

### TRIM50 Promotes the Sequestration of Ubiquitinated Proteins into Aggresome and Drives the Accumulation of p62 and HDAC6

We investigated whether TRIM50 and p62 colocalize into the aggresome. First, by fluorescence microscopy we showed that p62 colocalizes with HDAC6 upon MG132 treatment (Figure 4A). Then we found a partial colocalization between endogenous p62 and TRIM50 in SH-SY5Y cells (Figure 4B, a–c), which was intensified in presence of MG132 (Figure 4B, d–f).

Next we asked whether TRIM50 has any role in the recruitment and/or accumulation of polyubiquitinated proteins to the aggresome. MG132 treatment resulted in a prominent accumulation of higher-molecular-weight species constituted by polyubiquitinated proteins in the detergent insoluble fraction as showed by immunoblot with an anti-ubiquitin antibody (Figure 4C). These data were confirmed in *Trim50* deficient mouse embryo fibroblasts (kindly provided by Prof. Reymond). Depletion of endogenous Trim50 resulted in a decrease of polyubiquitinated protein levels in MG132 treated cells (compare lane 4 to 8 in Figure 4D). We then examined whether the TRIM50 depletion influences the area, perimeter, and intensity of FK2-polyubiquitinated dots. MEF *Trim50*−/− cells showed a significant decrease of signal intensity, compared to the wild type and heterozygous counterpart, while no significant difference was observed for the area and size of the dots (Figure 5A). Notably, MEF *Trim50*−/− cells treated with MG132 showed a highly significant reduction of all three parameters compared to the wild type and heterozygous mouse cell lines, respectively (Figure 5B).

Since the aggregates formation is a reversible process, to explore the effect of TRIM50 on the clearance of aggresome components, we analyzed the aggresome insolvency. MG132-pretreated MEF *Trim50* cells were incubated in a free-drug media for 48 h, and the FK2-aggresome positive cells were counted. Immediately after the removal of MG132, we found a significant decrease of the number of FK2-positive aggregates in *Trim50*−/− cells compared to the *Trim50*+/+ (Figure 5C). More interestingly, 48 hours after the MG132 removal we observed a significantly higher number of FK2-positive aggregates in MEF *Trim50*−/− compared to MEF *Trim50*+/+ suggesting that Trim50 is required for the clearance of polyubiquitinated proteins included within aggresome (p = 0.03, Figure 5C). Thus our analysis suggests that TRIM50 plays an active role in the sequestration of polyubiquitinated proteins in the aggresome.

Finally, we assessed whether TRIM50 overexpression has any effect on protein level of the endogenous HDAC6 and p62. p62 protein enrichment was observed in both soluble and insoluble fractions upon TRIM50 overexpression in the presence of MG132 (Figure S5A–B). Likewise we observed an increase of HDAC6 protein amount in both fractions, mainly upon MG132 treatment (Figure S5C). These results demonstrate that TRIM50 promotes the accumulation of both p62 and HDCA6, particularly into detergent insoluble aggregates.

### Inhibition of Lysosomal Activity Results in TRIM50 Accumulation and Increases the Number of TRIM50-positive Bodies

To evaluate the role of the autophagy in the turnover of TRIM50, we measured the amount of TRIM50 protein level in cells treated or not with the lysosomal enzyme inhibitor ammonium chloride (NH_4_Cl) (Figure 6A–B). Compared with DMSO treated-cells, treatment with NH_4_Cl resulted in about 5 fold-increases in TRIM50 levels over time. Moreover we monitored the subcellular localization of TRIM50 cytoplasmic bodies after induction and inhibition of autophagic flux. In cells treated with NH_4_Cl, we observed a slight but clear increase in the number of intracellular TRIM50 positive bodies (Figure 6 C). Notably, FK2 positive proteins lose their colocalization with TRIM50 bodies after the inhibition of autophagic flux. These data suggested that TRIM50 itself and TRIM50-related bodies might be mainly degraded by autophagy-lysosomal pathway.

## Discussion

In this study we showed that the E3 ubiquitin ligase TRIM50 forms highly dynamic and heterogeneous cytoplasmic bodies containing polyubiquitinated proteins. Inhibition of proteasome activity resulted in the coalescence of TRIM50 bodies into aggresome and in their colocalization with HDCA6 protein; this localization does not depend on the E3 ubiquitin ligase activity of TRIM50. Using fibroblast from *Hdac6*-deficient mice, we demonstrated that the TRIM50 aggresome localization is HDAC6-dependent. Importantly, in the presence of MG132, we observed that TRIM50 bodies change their shape in absence of HDAC6, becoming larger and lost the localization into the aggresome. Overall these evidences demonstrated that the TRIM50 inclusion bodies are aggresome precursors. Evidence that the TRIM50 localization is not merely artifact of overexpression comes from experiment in which the endogenous TRIM50 displayed a very similar localization to that of transfected protein (Figure 1B and S3B). Importantly this study demonstrated that TRIM50 is a novel component of and promotes the accumulation of ubiquitinated substrates to aggresome. Moreover we identified two novel TRIM50 partners, HDAC6 and p62, both involved in the clearance of polyubiquitinated and misfolded protein aggregates [2], [3], [4], [24].

The composition of the aggresome was partially solved by mass spectrometry [25], [26]. Song and colleagues showed that the higher proportion of aggresome-enriched proteins is related to molecular chaperones and ubiquitin-proteasome system components, involved in the elimination of misfolded and/or ubiquitinated proteins from cells [27]. Notably, a number of TRIM50 interactors that we have isolated in our proteomics approach, have been identified in a recent screening of proteins associated with MG132-induced aggresome in SH-SY5Y cells [26]. Among them are proteins known to interact with misfolded proteins and play a role in protein aggregation [28] including p62, chaperone proteins like Serpin H1, HSP90B1, PPIB, and 14-3-3 (eta and zeta) (Table S2). Moreover a number of the TRIM50-bound proteins were found ubiquitinated in previous studies [29], [30] or annotated in the Ubiprot database [31] (Table S2). Overall these data give additional evidences that TRIM50 bodies are aggresome precursors involved in the ubiquitination and aggregation process of misfolded proteins.

We confirmed the interaction of TRIM50 with p62. p62 is a multifunctional adapter protein implicated in autophagy, cell signaling, receptor internalization, inflammation and protein turnover [22]. p62 is found in cytosolic protein aggregates that accumulate in various chronic, toxic, and degenerative diseases. It interacts with ubiquitinated proteins carrying them on the road to autophagy-mediated degradation [23], [32]. The TRIM50-p62 interacting region involves amino acids 302–370, a region that includes the LC3-Interacting Region (LIR) domain involved in the binding to LC3 (microtubule-associated protein 1A/1B light chain 3) [23] a modifier protein that plays a pivotal role in autophagosome biogenesis [33]. Interestingly we have some preliminary data showing that TRIM50 and LC3 colocalize in both normal and autophagy-induced conditions (Figure S4B and Fusco, unpublished results). Moreover using the NH_4_Cl autophagy inhibitor, we found that TRIM50 is partially degraded through the autophagy-lysosomal pathway (Figure 6). However how this degradation occurs remains yet unclear; one possibility is that TRIM50 could directly associate with LC3, or that TRIM50 could be addressed together with p62 to autophagy machinery for its degradation. In that way the observed colocalization between TRIM50 and LC3 is intriguing and deserves more investigations. These findings suggest also that p62 may serve as a scaffold protein, via the interaction with TRIM50, whereby chains of polyubiquitin are transferred to target substrates for degradation. Nevertheless it is tempting to speculate that TRIM50 might be involved in autophagy processes as well.

Increasing evidences indicate that autophagy-related proteins are sequestrated into the aggresome as a selective mechanism to regulate their degradation [24]. Since aggresome formation mainly takes place in the insoluble fraction [34], [35] we assessed whether TRIM50 has a role in the accumulation of polyubiquitinted proteins. We observed that TRIM50 promotes the recruitment of polyubiquitinated proteins to aggresome and that the observed decrease of aggresome clearance was associated to the depletion of TRIM50 (Figure 5C), suggesting that these proteins are TRIM50 substrates.

Overall the data reported in this study reveal a role for TRIM50 in aggresome formation and add further insights on its function by identifying and characterizing its first two protein partners. We speculate that, when the proteasome activity is impaired, TRIM50 ensures the sequestration of its targets to the aggresome via the association with HDAC6 and their subsequent likely removal by p62-mediated autophagy. Further studies, particularly the identification of TRIM50 specific substrates, are needed to unequivocally assess the authenticity of this model.

Accumulation of polyubiquitinated protein aggregates is a hallmark of several neurodegenerative disorders as well as of a number of other protein aggregation diseases affecting muscles, heart, liver and lung [36], [37]. p62 has been identified as a component of inclusion bodies in several human diseases, such as neurodegenerative diseases (e.g., Alzheimer’s disease, Parkinson’s disease, and amyotrophic lateral sclerosis) and in liver diseases (e.g., alcoholic hepatitis, hepatic steatosis, and hepatocellular carcinoma) [38]. It will hence be interesting to investigate whether TRIM50 is also a component of such bodies and it could even be responsible for targeting p62 to these sites.

## Materials and Methods

### Fusion Plasmids

The pcDNA3-EGFP and pCDNA3-HA wild-type and mutants TRIM50 were described in [15]. Human β2-tubulin ORF was cloned into a pcDNA3 vector with FLAG as tag using a PCR based method with appropriate oligonucleotides followed by in-frame insertion into the vector. DsRED -LC3 and pENTR-EGFP, GST tagged p62 and GST-p62 mutants were a kind gift from Prof. T. Johansen (Institute of Medical Biology, University of Tromso, Norway), pcDNA3-Myc-p62 was a generous gift of Prof. Marie W. Wooten (Cellular and Molecular Biosciences Program, Auburn University, USA). pcDNA3-HA-HDAC6 mutants were a gift of Prof. Matthias (Friedrich Miescher Institute for Biomedical Research, Basel, Switzerland). The plasmids used in this study are listed in Table S1.

### Cell Culture and Stable Cell Line Production

HEK293, HeLa cells, SH-SY5Y (all from ATCC, Manassas, USA), and MEF were maintained in DMEM with Glutamax medium supplemented with 10% fetal bovine serum and 1% antibiotics (Invitrogen, Carlsbad, CA). Hdac6 MEF (mouse embryo fibroblast) cells were kindly provided by Prof. Joo-Yong Lee (Duke University, Durham, USA) [39].

Fugene 6 (Roche) was used for transfection according to the manufacturers’ instructions. HEK293 were transfected with pcDNA3-FLAG-TRIM50 or empty vector and selected for 2 weeks with 1 mg/ml G418 (Invitrogen, Carlsbad, CA) selective agent. The expressing colonies were expanded and then used for protein extract preparations following standard procedures. HEK293 cell line was used since the low level expression of endogenous TRIM50 protein.

Hereafter the stable cell lines will be referred to as FLAG-TRIM50#3 and FLAG#3, respectively.

### Mouse Embryo Fibroblast Generation

To identify loss-of-function mutation in *Trim50*, we screened the sequence of the first exon of this gene in the sperm DNA archives of F1 male progeny of ENU-treated (ethylnitrosourea) males and untreated females established by INGENIUM (http://www.ingenium-ag.com). We identified two missense (V55M and V60G) and one nonsense mutation. The latter mutation creates a premature stop codon by modifying TGC into a TGA amber codon. This C52X mutation in the middle of the RING domain abrogates the E3-ligase activity of Trim50, as the next in frame methionine residue is situated in the coiled-coil domain after both the RING and the B-box type 2 of the *Trim50*-encoded protein. We recovered the nonsense mutant mouse from the frozen archive using *in vitro* fertilization. Due to the generation method, the first heterozygote mice (F1) have around twenty “background” mutations in addition to the wanted one. They were backcrossed with C3HeB/FeJ wild-type mice from Jackson laboratories for 12 generations to purge the strain of other mutations potentially induced by the ENU treatment. Mouse embryonic fibroblasts (MEFs) were prepared from E13.5 embryos as described in [40]. Cells were subsequently genotyped by Sanger sequencing to identify +/+, +/− and −/− lines. All procedures used with mice models and to generate the MEFs were approved by the CIG Institutional Animal Care and followed the National Institutes of Health Guidelines, ‘Using Animals in Intramural Research’. The work was approved by the ethics committee of the Veterinarian Cantonal Office (authorization Vaud-1958).

### Immunofluorescence Microscopy

For immunofluorescence analyses, the cells transfected with EGFP-TRIM50 were fixed before their incubation with the primary and secondary antibodies of interest, mounted in mowiol and examined on a Zeiss LSM 510 META confocal microscope (Carl Zeiss, Jena, Germany). All confocal images were obtained using the necessary filter sets for GFP, Alexafluor 488 and 546, using a Zeiss Plan-Neofluor 63× oil immersion objective (NA 1.4), with the pinhole set to one Airey unit.

### Live Cell Imaging and Fluorescent Recovery After Photobleaching (FRAP) Analyses

HeLa cells were transfected with EGFP-TRIM50 construct and observed at 37°C in 20mM HEPES buffered DMEM using a Zeiss LSM 510 META confocal microscope (Carl Zeiss, Jena, Germany). Temperature was controlled with a Nevtek air stream stage incubator (Burnsville, VA, USA) and images of live cells were acquired using sequential excitation at 488 nm and 543 nm. The tracking of moving objects and evaluation of their speed were performed using the Tracking macro of the ImageJ program. Selective photobleaching in the regions of interest within the cell was carried out on the Zeiss LSM510 using 100 iterations with a 488 nm laser line at full power.

### Correlative Light Electron Microscopy (CLEM)

EGFP-TRIM50 transfected HeLa cells were grown on CELLocate coverslips with coordinated grid and then prepared for CLEM microscopy identification of TRIM50 structures according to Polishchuk et al. [41]. Briefly, after visualization of EGFP-TRIM50 positive bodies by time-lapse confocal microscopy, the cells were fixed, labeled with an antibody against EGFP using the gold-enhance protocol, embedded in Epon-812, and cut in serial sections. Then region containing TRIM50-positive bodies were analyzed in serial thin sections under a Philips Tecnai-12 electron microscope (Philips, Einhoven, The Netherlands). EM images were acquired from the region of interest using an Ultra View CCD digital camera (Soft Imaging Systems, Munich, Germany).

### Statistical Analysis

All microscopy experiments were performed in triplicate. Approximately 40 cells were analyzed for each experimental condition. For the immunofluorescence experiments on Trim50-MEF cells, area, perimeter, and intensity of FK2-positive dots were measured using ImageJ program. The threshold was set to a level that excludes all the cytosolic background, thus allowing selective analysis of intense puncta representing the FK2-positive dots. Data were reported as median along with the upper and lower quartiles (Q_1_–Q_3_). Normal distribution assumption was checked by means of Q-Q plot, Shapiro-Wilks and Kolmogorov-Smirnov tests. The followed parameters were log transformed before statistical analyses because of their skewed distribution. Comparisons between wild-type, heterozygous and knockout mice were assessed by means of specific contrasts defined into a hierarchical linear model (HLM), accounting for clustering due to multiple measures collected within cell. All p-values were adjusted for multiple comparison following Tukey-Kramer’s method. A p-value <0.05 was considered for statistical significance. All statistical analyses and graphs were performed using SAS Release 9.1 (SAS Institute, Cary, NC, USA) and R (version 2.10.1) software, respectively.

### Protein Identification by Mass Spectrometry Analysis

TRIM50 complexes were isolated from HEK293 cells total extracts by immunoprecipitation. FLAG-TRIM50#3 and FLAG#3 cell lines were lysed in PBS, 0.5% NP-40, 1 mM PMSF, and COMPLETE protease inhibitors (Roche) for 45 min under gently mixed. Total protein extracts were pre-cleared with unspecific Mouse IgG Agarose Beads (Sigma) overnight in lysis buffer. The protein extracts were recovered by centrifugation (3000 rpm for 5 min) and then incubated overnight, under gently agitation, onto M2 anti-FLAG agarose-conjugated antibody beads (Sigma) previously blocked with no fat milk treatment. Unbound proteins were discarded and the beads were collected by centrifugation and extensively washed with lysis buffer supplemented with 150 mM NaCl to eliminate non-specific bound proteins. Elution of the desired protein complexes was performed by competition with FLAG peptide in elution buffer. The eluted proteins were precipitated in methanol/chloroform and then loaded onto a 10% SDS-PAGE. The gel was stained with colloidal Coomassie blue (Pierce). Protein bands were excised from the gel, reduced, alkylated and digested with trypsin as described elsewhere (Zito et al., 2007). Peptide mixtures extracted from the gel were analyzed by nano-chromatography tandem mass spectrometry (nanoLC–MS/MS) on a CHIP MS Ion Trap XCT Ultra equipped with a capillary 1100 HPLC system and a chip cube (Agilent Technologies, Palo Alto, CA). Peptide analysis was performed using data-dependent acquisition of one MS scan (mass range from 400 to 2000 m/z) followed by MS/MS scans of the three most abundant ions in each MS scan. Raw data from nanoLC–MS/MS analyses were employed to query a non-redundant protein database using in house MASCOT software (Matrix Science, Boston, USA).

### Immunoprecipitation, GST Pull-down and Western Blot

Co-immunoprecipitation experiments were performed as previously described [42]. Complexes were analyzed by western blotting using indicated antibodies. Horseradish peroxidase conjugated anti-mouse and anti-rabbit antibodies (GE Healthcare) and the ECL chemiluminescence system (GE Healthcare) was used for detection. Where indicated the MG132 proteasome inhibitor (Calbiochem, USA) was added. GST-p62 (231–385) and GST-p62 (232–370) fusion proteins were purified using glutathione-Sepharose 4B beads (GE Healthcare) according to the manufacturer’s instructions. For the GST pulldown assay, 3 µg of GST-recombinant proteins were mixed with 40 µg of total FLAG-TRIM50#3 cell lysate and incubated at 4°C for 2 h with rotation, and then incubated with FLAG antibody for 4 h. The binding fraction was washed four times and then loaded into a SDS 10% PAGE gel, and immunoblotted with anti-GST antibody (Santa Cruz). Soluble and insoluble fractions were obtained using RIPA buffer as described elsewhere (Muqit et al., 2006). Protein band densities were determined using densitometer (Kodak). The amount of the protein was calculated by the initial amount of FLAG-protein level and normalized with GAPDH. Cells treated with DMSO were used as control.

## Supporting Information

Figure S1 **TRIM50 does not colocalize with known and induced cellular organelles.** (A) HeLa cells were transiently transfected with EGFP-TRIM50, fixed and stained for different cytoplasmic markers. The panel shows the merge of EGFP-TRIM50 (green) with single marker (red): Golgi markers (a,b,c,d,i), early endosomes (e), the coat complex of endoplasmic reticulum associated vesicles (f,g,h), lysosomes (j), cytoskeletal structures (k), and peroxisomes (l). (B) HeLa cells were transiently transfected with EGFP-TRIM50. After 24h the cells were cultured in presence of arsenite (0.5 mM for 30 min.) before processed and immunostained with anti-Eif3 for stress granules (b,c), and anti-HuR for P-bodies (e,f).
(TIF)

Figure S2 **Movement of TRIM50 particles and determination of the movement rates.** (A) HeLa cells transiently expressing EGFP-TRIM50 were imaged over 110 frames at 0.5 sec intervals. The individual frames were summed using ImageJ. The tracks of three single particles are shown (blue, particle a; green, particle b; red, particle c). The asterisks indicate starting point of each particle. (B) Velocities of particles a–c were measured throughout the time that they were observed during the frames by using manual tracking plugin of ImageJ program. The vertical Y-axis shows the velocity of particles in each frame (micron/sec); the horizontal X-axis shows the relative time during the image sequence in which the particle was observed. (C) Recovery of the signal was measured throughout the time observed during 221 frames sequence using manual tracking of ImageJ program. (D) HeLa cells were transfected with EGFP-TRIM50, incubated for 30 min with TRITC-dextran, and imaged using confocal fluorescence microscopy. EM immunogold-labeled of a section corresponding to the area indicated by the box in figure D.
(TIF)

Figure S3 **TRIM50 interacts with beta tubulin.** (A) Endogenous TRIM50 colocalizes with polyubiquitinated proteins. SH-SY5Y cells were stained with anti-TRIM50 antibody and with FK2 antibody. Where indicated, the cells were treated with 25 µM MG132 for 6 h. (B) HeLa cells expressing EGFP-TRIM50 were stained with an anti-HDAC6 antibody. The cells were treated with 25 µM MG132 for 6 h (d–f). (C) The interaction between TRIM50 and beta tubulin was assayed in HEK293 cells transiently expressing FLAG-Tubb2b and EGFP-TRIM50. The cell lysates were immunoprecipitated with anti-FLAG and immunoblotted with anti-GFP antibody.
(TIF)

Figure S4 **(A) TRIM50 E3-ubiquitin ligase activity is not required for aggresome localization.** MEF *Trim50*−/− cell line was transfected with EGFP-Trim50ΔRING, treated with 25 µM MG132 for 6 h and stained with HDAC6 antibody (a–f). (B) TRIM50 colocalizes with LC3. HeLa cells were transiently co-transfected with HA-TRIM50 and DsRed-LC3 and stained with anti-HA antibody. After 24h the cells were incubated with EBSS medium for 2 h (d–f).
(TIF)

Figure S5 **TRIM50 increases the insolubility of p62 and HDAC6 into the aggresome.** (A–B–C) TRIM50 promotes the sequestration of p62 and HDAC6 in aggresome. Lysates from FLAG-TRIM50#3 and FLAG#3 cell lines, treated with vehicle (–) or with MG132 (+) were separated in detergent-soluble and detergent insoluble fractions and immunoblotting with anti-FLAG and anti-p62 antibodies. An example for p62 protein is depicted (A). The asterisk shows the relative band of endogenous p62 of previous immunoblotting. The relative level of soluble and insoluble fractions was measured by quantification of the intensity of p62 (B) and HDAC6 (C) bands of three indipendent experiments.
(TIF)

Movie S1 **EGFP-TRIM50 bodies movement. Movies show a 110 frames sequence captured at 0.5 second intervals.**
(MOV)

Movie S2 **FRAP analysis of TRIM50 cytoplasmic bodies. Movies show a 224 frames sequence captured at 0.5 second intervals.**
(MOV)

Table S1 **Plasmids used in this study.**
(DOC)

Table S2 **TRIM50-associated proteins found ubiquitinated and/or present within induced Aggresome.**
(DOC)
