# Supplementary figures and images for: The E3-Ubiquitin Ligase TRIM50 Interacts with HDAC6 and p62, and Promotes the Sequestration and Clearance of Ubiquitinated Proteins into the Aggresome
Source: PLoS One. 2012 Jul 9;7(7):e40440. doi: 10.1371/journal.pone.0040440 (PMC3392214; doi:10.1371/journal.pone.0040440)

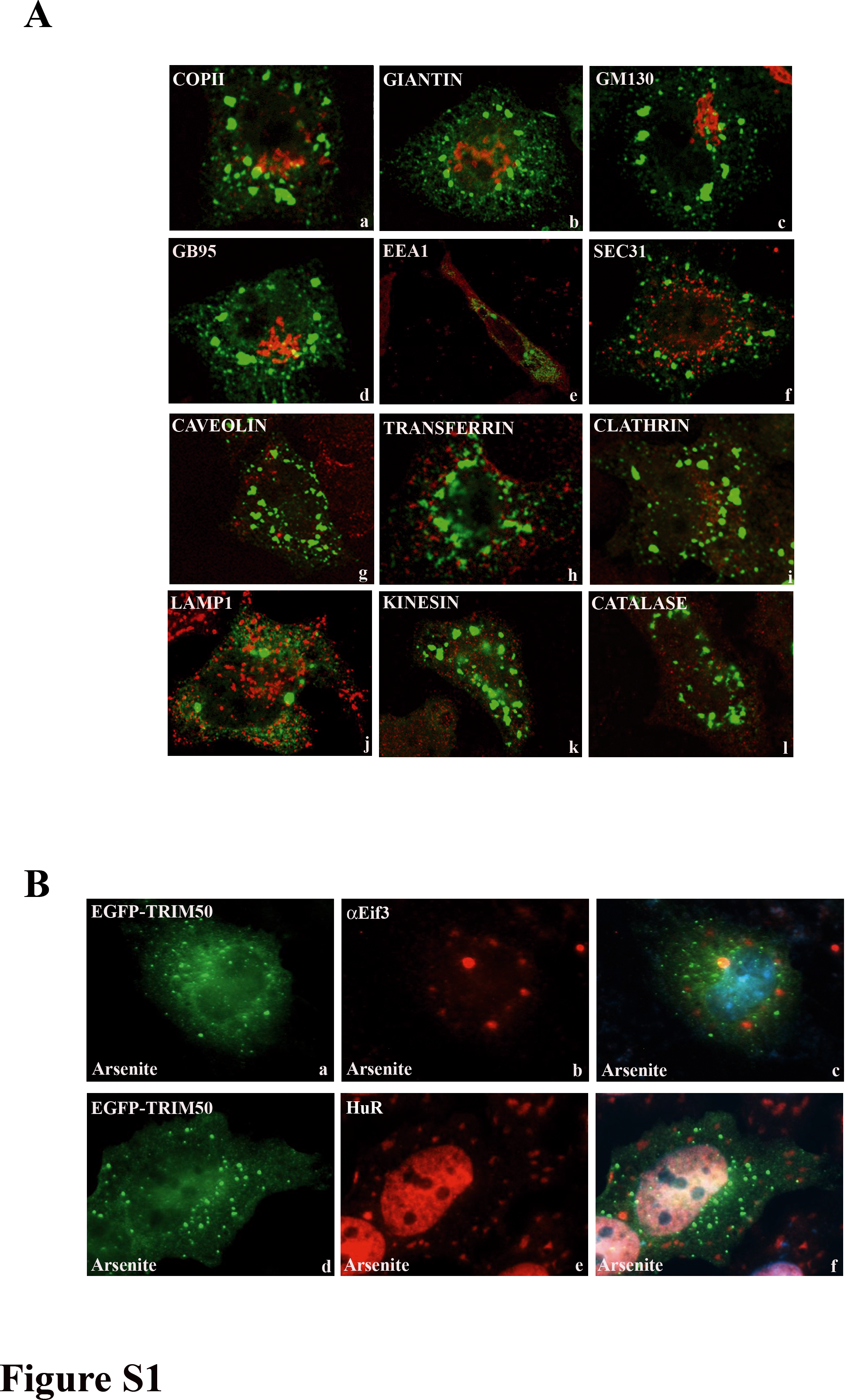

Supplement: Figure S1 — TRIM50 does not colocalize with known and induced cellular organelles. (A) HeLa cells were transiently transfected with EGFP-TRIM50, fixed and stained for different cytoplasmic markers. The panel shows the merge of EGFP-TRIM50 (green) with single marker (red): Golgi markers (a,b,c,d,i), early endosomes (e), the coat complex of endoplasmic reticulum associated vesicles (f,g,h), lysosomes (j), cytoskeletal structures (k), and peroxisomes (l). (B) HeLa cells were transiently transfected with EGFP-TRIM50. After 24h the cells were cultured in presence of arsenite (0.5 mM for 30 min.) before processed and immunostained with anti-Eif3 for stress granules (b,c), and anti-HuR for P-bodies (e,f). (TIF) [file pone.0040440.s001.tif]

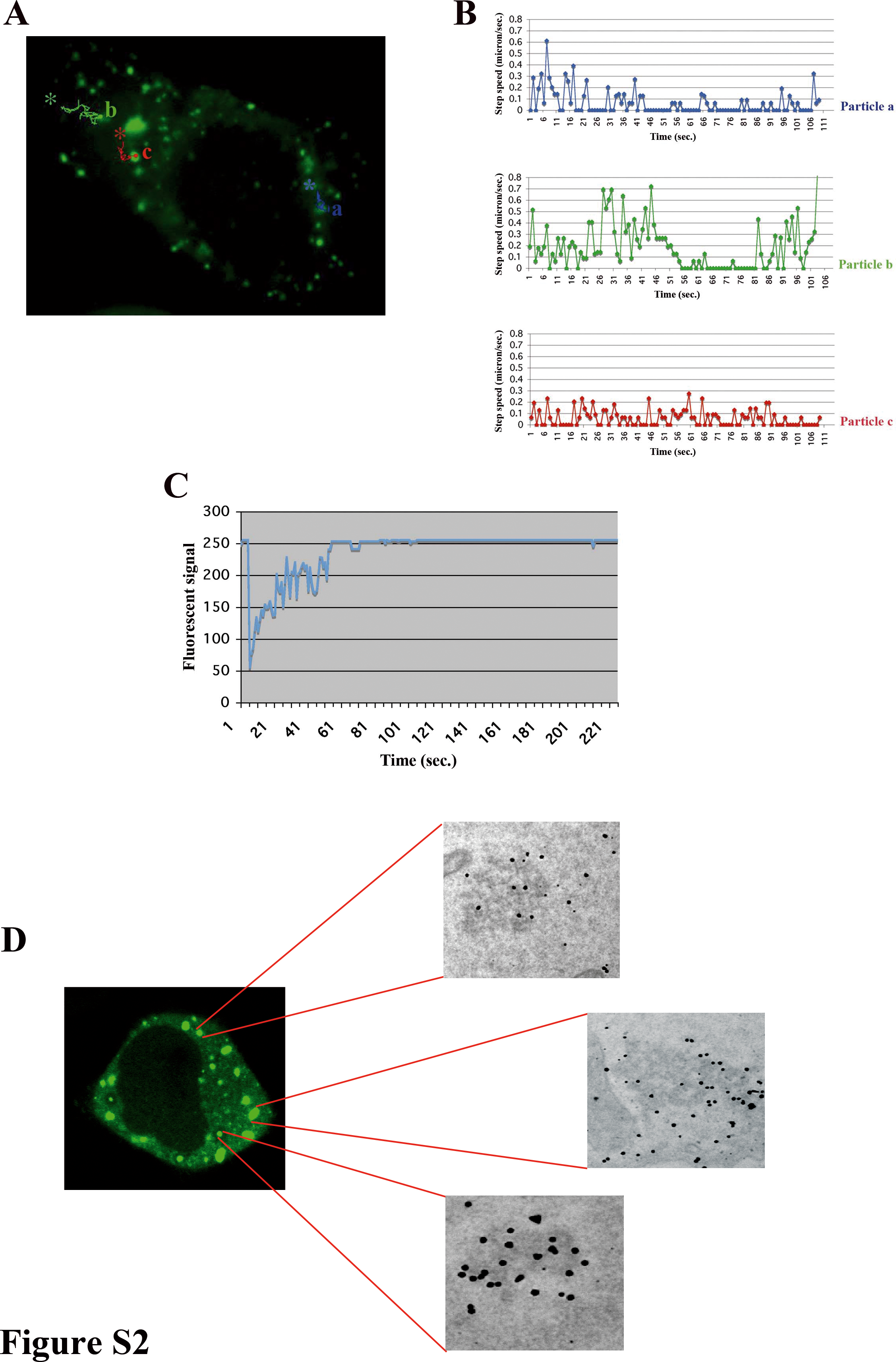

Supplement: Figure S2 — Movement of TRIM50 particles and determination of the movement rates. (A) HeLa cells transiently expressing EGFP-TRIM50 were imaged over 110 frames at 0.5 sec intervals. The individual frames were summed using ImageJ. The tracks of three single particles are shown (blue, particle a; green, particle b; red, particle c). The asterisks indicate starting point of each particle. (B) Velocities of particles a–c were measured throughout the time that they were observed during the frames by using manual tracking plugin of ImageJ program. The vertical Y-axis shows the velocity of particles in each frame (micron/sec); the horizontal X-axis shows the relative time during the image sequence in which the particle was observed. (C) Recovery of the signal was measured throughout the time observed during 221 frames sequence using manual tracking of ImageJ program. (D) HeLa cells were transfected with EGFP-TRIM50, incubated for 30 min with TRITC-dextran, and imaged using confocal fluorescence microscopy. EM immunogold-labeled of a section corresponding to the area indicated by the box in figure D. (TIF) [file pone.0040440.s002.tif]

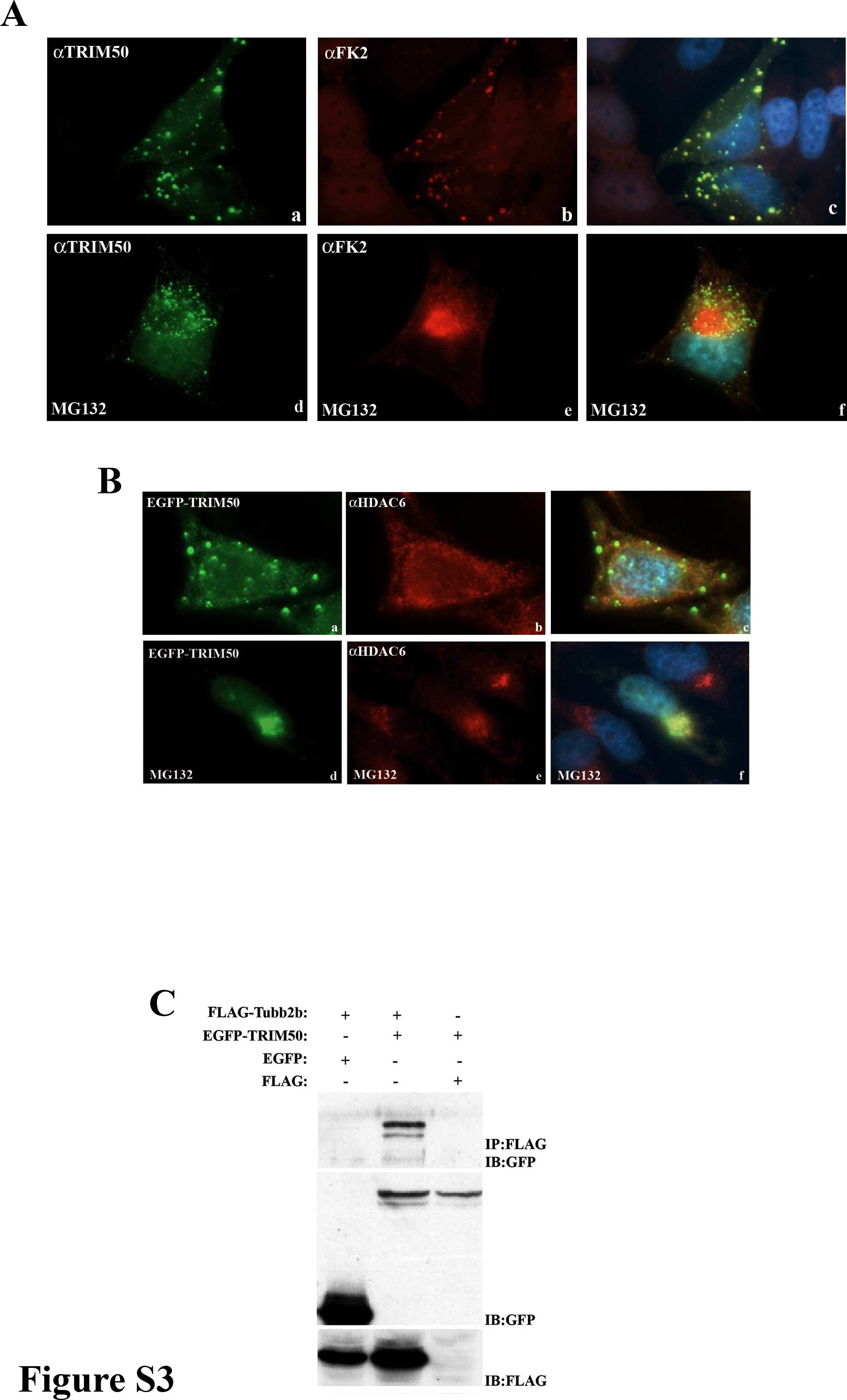

Supplement: Figure S3 — TRIM50 interacts with beta tubulin. (A) Endogenous TRIM50 colocalizes with polyubiquitinated proteins. SH-SY5Y cells were stained with anti-TRIM50 antibody and with FK2 antibody. Where indicated, the cells were treated with 25 µM MG132 for 6 h. (B) HeLa cells expressing EGFP-TRIM50 were stained with an anti-HDAC6 antibody. The cells were treated with 25 µM MG132 for 6 h (d–f). (C) The interaction between TRIM50 and beta tubulin was assayed in HEK293 cells transiently expressing FLAG-Tubb2b and EGFP-TRIM50. The cell lysates were immunoprecipitated with anti-FLAG and immunoblotted with anti-GFP antibody. (TIF) [file pone.0040440.s003.tif]

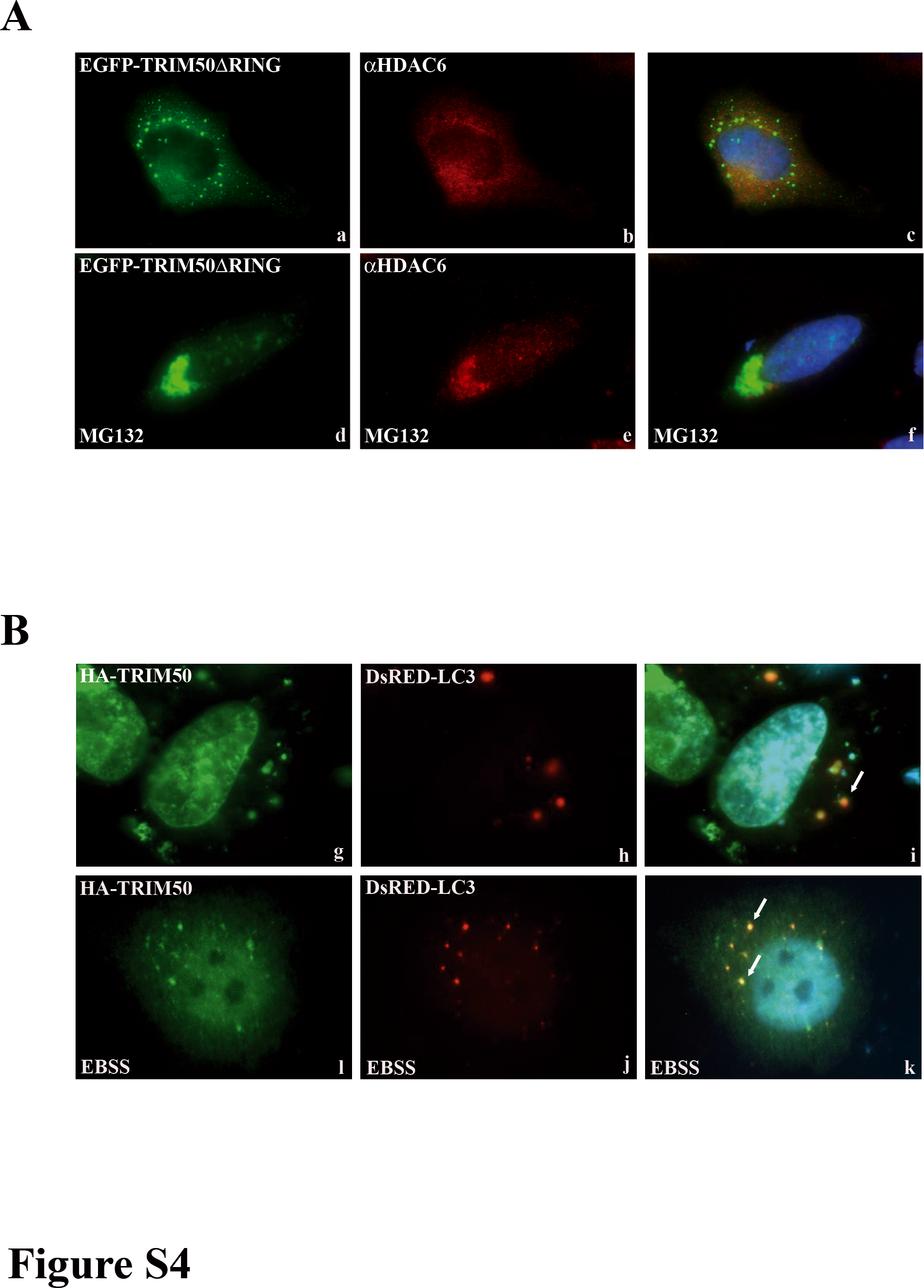

Supplement: Figure S4 — (A) TRIM50 E3-ubiquitin ligase activity is not required for aggresome localization. MEF Trim50−/− cell line was transfected with EGFP-Trim50ΔRING, treated with 25 µM MG132 for 6 h and stained with HDAC6 antibody (a–f). (B) TRIM50 colocalizes with LC3. HeLa cells were transiently co-transfected with HA-TRIM50 and DsRed-LC3 and stained with anti-HA antibody. After 24h the cells were incubated with EBSS medium for 2 h (d–f). (TIF) [file pone.0040440.s004.tif]

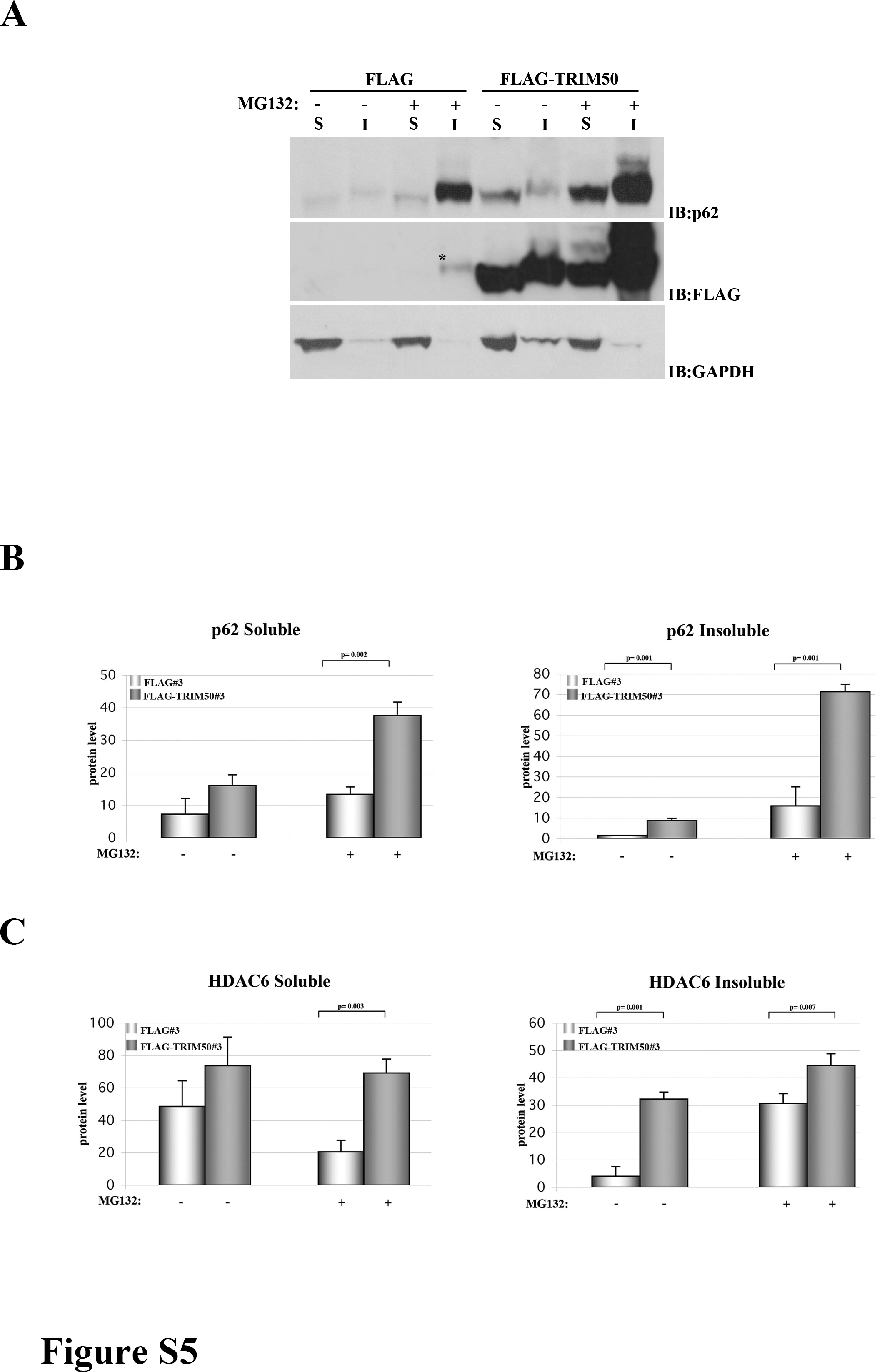

Supplement: Figure S5 — TRIM50 increases the insolubility of p62 and HDAC6 into the aggresome. (A–B–C) TRIM50 promotes the sequestration of p62 and HDAC6 in aggresome. Lysates from FLAG-TRIM50#3 and FLAG#3 cell lines, treated with vehicle (–) or with MG132 (+) were separated in detergent-soluble and detergent insoluble fractions and immunoblotting with anti-FLAG and anti-p62 antibodies. An example for p62 protein is depicted (A). The asterisk shows the relative band of endogenous p62 of previous immunoblotting. The relative level of soluble and insoluble fractions was measured by quantification of the intensity of p62 (B) and HDAC6 (C) bands of three indipendent experiments. (TIF) [file pone.0040440.s005.tif]
