# Supplementary material for: The E3-Ubiquitin Ligase TRIM50 Interacts with HDAC6 and p62, and Promotes the Sequestration and Clearance of Ubiquitinated Proteins into the Aggresome
Source: PLoS One. 2012 Jul 9;7(7):e40440. doi: 10.1371/journal.pone.0040440 (PMC3392214; doi:10.1371/journal.pone.0040440)
Supplement: Table S1 — Plasmids used in this study. (DOC) [file pone.0040440.s008.doc]

**Table S1: Plasmids used in this study**

| **Vectors** | **Description** | **Source** |
| --- | --- | --- |
| pCDNA3-EGFP-Myc | Mammalian EGFP fusion expression vector; CMV promoter | this study |
| pCDNA3-FLAG | Mammalian EGFP fusion expression vector; CMV promoter | this study |
| pCDNA3-HA | Mammalian EGFP fusion expression vector; CMV promoter | this study |
|  |  |  |
| *constructs made by traditional cloning or site-directed mutagenesis* | | |
|  |  |  |
| pCDNA3-EGFP-Myc-TRIM50 | TRIM50 in pCDNA3 vector | this study |
| pCDNA3-HA-TRIM50 | TRIM50 in pCDNA3 vector | this study |
| pCDNA3-FLAG-TRIM50 | TRIM50 in pCDNA3 vector | this study |
| pCDNA3-Myc-TRIM50 | TRIM50 in pCDNA3 vector | this study |
| p-YFP-C1-TRIM50 | TRIM50 in p-YFP vector | this study |
| pGEX 4T1-TRIM50 | TRIM50 in p-GEX vector | this study |
| pcDNA3-TRIM50 | TRIM50 in pCDNA3 vector | this study |
| pcDNA3-FLAG-TRIM50-ΔCoil1 | TRIM50 with Coil_1 deletion in pCDNA3 vector | this study |
| pcDNA3-FLAG-TRIM50 ΔCoil2 | TRIM50 with Coil_2 deletion in pCDNA3 vector | this study |
| pcDNA3-FLAG-TRIM50 ΔCoil1/2 | TRIM50 with Coil_1-2 deletion in pCDNA3 vector | this study |
| pCDNA3-EGFP-Myc-ΔRING_TRIM50 | TRIM50 domain (73-483) in pCDNA3 vector | this study |
| pCDNA3-FLAG-ΔRING_TRIM50 | TRIM50 domain (73-483) in pCDNA3 vector | this study |
| pCDNA3-EGFP-Myc-RING_TRIM50 | TRIM50 domain (1-74) in pCDNA3 vector | this study |
| pCDNA3-EGFP-FLAG-RING_TRIM50 | TRIM50 domain (1-74) in pCDNA3 vector | this study |
| pCDNA3-EGFP-Myc-ΔRFP_TRIM50 | TRIM50 domain (1-270) in pCDNA3 vector | this study |
| pCDNA3-FLAG-ΔRFP_TRIM50 | TRIM50 domain (1-270) in pCDNA3 vector | this study |
| pCDNA3-EGFP-Myc-RFP_TRIM50 | TRIM50 domain (270-483) in pCDNA3 vector | this study |
| pCDNA3-FLAG-RFP_TRIM50 | TRIM50 domain (270-483) in pCDNA3 vector | this study |
| pCDNA3-EGFP-Myc-Bb-Cc_TRIM50 | TRIM50 domain (73-270) in pCDNA3 vector | this study |
| pCDNA3-FLAG-Bb-Cc_TRIM50 | TRIM50 domain (73-270) in pCDNA3 vector | this study |
| pCDNA3-Myc-p62 | p62 domain in pCDNA3 vector | Dr. M. Wooten |
| pGEX 4T1-GST-p62 (232-370) | p62 domain in p-GEX vector | this study |
| pGEX 4T1-GST-p62 (231-385) | p62 domain in p-GEX vector | this study |
| pEGFP-C1-p62 | p62 in pEGFP-C1 vector | Dr. T. Johansen |
| pEGFP-C1-p62 (170-302) | p62 domain in pEGFP-C1 vector | Dr. T. Johansen |
| pEGFP-C1-p62 (124-256) | p62 domain in pEGFP-C1 vector | Dr. T. Johansen |
| pEGFP-C1-p62 (1-385) | p62 domain in pEGFP-C1 vector | Dr. T. Johansen |
| pEGFP-C1-p62 (122-440) | p62 domain in pEGFP-C1 vector | Dr. T. Johansen |
| pEGFP-C1-p62 (123-385) | p62 domain in pEGFP-C1 vector | Dr. T. Johansen |
| Ds-Red-LC3 | LC3 in Ds-Red vector | Dr. T. Johansen |
| pCDNA3-FLAG-HDAC6 | HDAC6 in pCDNA3 vector | Dr. Joo-Yong |
| pCDNA3-FLAG-ΔBUZ_HDAC6 | HDAC6 domain in pCDNA3 vector | Dr. Joo-Yong |
| pCDNA3-FLAG-HDAC6 (1-503) | HDAC6 domain in pCDNA3 vector | Dr. Joo-Yong |
| pCDNA3-HA-CAT2_HDAC6 | HDAC6 domain in pCDNA3 vector | Dr. P.Matthias |
| pCDNA3-HA-C_HDAC6 | HDAC6 domain in pCDNA3 vector | Dr. P.Matthias |
