# Supplementary material for: The E3-Ubiquitin Ligase TRIM50 Interacts with HDAC6 and p62, and Promotes the Sequestration and Clearance of Ubiquitinated Proteins into the Aggresome
Source: PLoS One. 2012 Jul 9;7(7):e40440. doi: 10.1371/journal.pone.0040440 (PMC3392214; doi:10.1371/journal.pone.0040440)
Supplement: Table S2 — TRIM50-associated proteins found ubiquitinated and/or present within induced Aggresome. (DOC) [file pone.0040440.s009.doc]

| LC–MS/MS_TRIM50 | SwissProt | Wilde, 2011 | UbiProt data  Chernorudskiy,  2007 | Meierhofer,  2008 | Vasilescu,  2005 |
| --- | --- | --- | --- | --- | --- |
| p62  Zinc finger protein 326 | P50454  Q5BKZ1 | +  + |  |  |  |
| WD repeat-containing P 68 | P61962 | + |  |  |  |
| UPF0027 protein C22orf28 | Q9Y3I0 | + |  |  |  |
| U5 snRNP component | Q15029 | + |  |  |  |
| U5 small NRP 200 kDa helicase | O75643 |  |  | + |  |
| Trifunctional Purine biosynthetic Padenosine-3 | P22102 |  |  | + |  |
| Serpin H1 | P50454 | + | + |  |  |
| Protein SEC13 homolog | P55735 |  | + |  |  |
| Peroxiredoxin-6 | P30041 | + |  |  |  |
| Pericentriolar material 1 protein | Q15154 | + |  |  |  |
| Peptidyl-prolylcis-trans isomerase B | P23284 | + |  |  |  |
| Nucleolar protein 5 | Q9Y2X3 | + |  |  |  |
| Nuclear pore complex Nup93 | Q8N1F7 | + |  |  |  |
| Matrin 3 | P43243 |  |  | + |  |
| L-lactate dehydrogenase B chain | P07195 | + |  |  |  |
| Leucine-rich PPR motif-containing protein Mit | P42704 |  |  | + |  |
| Glutaminyl-tRNA synthetase | P47897 | + |  |  |  |
| Filamin A | Q60FE6 | + |  | + |  |
| Eukaryotic translation initiation factor 3 | P60228 |  |  | + |  |
| Endoplasmin | P14625 | + |  | + | + |
| DNA-directed RNA polymerase II sub RPB2 | P30876 |  | + |  |  |
| Developmentally-regulated GTP-binding P 1 | Q9Y295 |  | + |  |  |
| Coatomer subunit beta' | P35606 | + |  | + |  |
| ATP-dependent RNA helicase DHX15 | O43143 |  |  | + |  |
| Apoptosis-inducing factor 1 | O95831 |  |  | + |  |
| 60S ribosomal protein L32 | P62910 |  | + |  |  |
| 26S proteasome regulatory sub 2 | Q13200 | + |  | + |  |
| 14-3-3 protein eta | Q04917 |  | + |  |  |
| 14-3-3 protein zeta | P63104 |  | + |  |  |

**Table S2: TRIM50-associated proteins found ubiquitinated and/or present within induced Aggresome**
